# Using the Behaviour Change Wheel and Theoretical Domains Framework to identify factors related to increasing fruit and vegetable consumption in children to guide future intervention development

**DOI:** 10.1017/S1368980024002362

**Published:** 2024-12-05

**Authors:** Louise George, Jenny Davison

**Affiliations:** School of Psychology, Ulster University, Coleraine BT52 1SA, UK

**Keywords:** COM-B model, BCW, Behaviour Change Wheel, Fruit and vegetables, TDF, Parents, Children, Qualitative, Behavioural diagnosis

## Abstract

**Objective:**

This study used the Behaviour Change Wheel (BCW) and Theoretical Domains Framework (TDF) to identify parental factors that are associated with increasing their child’s fruit and vegetable consumption. The information gathered enabled a behavioural diagnosis and the identification of intervention functions to increase fruit and vegetable consumption in children.

**Design:**

A qualitative design using open-ended online survey methodology was utilised.

**Setting:**

United Kingdom.

**Participants:**

Twenty-eight parents of primary school-aged children (4–11 years) aged 29–51 years participated.

**Results:**

Thematic and summative analysis identified skills in preparation and cooking, awareness of and desire to increase fruit and vegetable intake, knowledge of the recommendations and better health for their child as the main facilitators. The main barriers were time and financial constraints, their child’s food preferences and refusal to eat fruit and vegetables, negative role modelling from parents and grandparents and beliefs that fruit and vegetable intake will increase with age. For behaviour change to occur, ‘knowledge’, ‘social influences’, ‘environmental context and resources’, ‘beliefs about consequences’ and ‘beliefs about capabilities’ need to be altered.

**Conclusions:**

Novel findings suggest that future intervention development should focus on parental beliefs and skills around how to increase fruit and vegetable consumption as their child ages and expanding parental knowledge on the benefits of fruit and vegetable consumption such as mental and future health. The use of the TDF and BCW identified appropriate intervention functions that will guide future behaviour change techniques, modes of delivery and policy categories that best target increasing children’s fruit and vegetable consumption.

Fruit and vegetable consumption is a key part of a healthy balanced diet, as they contain essential vitamins, minerals and dietary fibre^(1)^. The WHO recommends that children from the age of 2 years consume at least five portions of fruit and vegetables daily^(2)^. In 2018, only 18 % of children aged 5–15 years in England ate five portions of fruit and vegetables daily^(3)^. It was reported that the average number of portions that children were eating was three portions per d^(3)^. Alarmingly, in Northern Ireland, consumption of five portions of fruit and vegetables is below the recommendation in all age groups^(4)^.

High fruit and vegetable intake is associated with physical health benefits such as reduced risk of cancer and CVD^(5,6)^ and low fruit and vegetable intake has been associated with a higher risk of obesity in school-aged children^(7)^. Fruit and vegetable intake has also been shown to be associated with greater respiratory health and children who consumed a higher intake of fruit at the age of 8 years had a lower risk of asthma which lasted up to the age of 24 years^(8)^. Fruit and vegetable intake is therefore essential for physical health. For every additional portion of fruit and vegetable intake, it has been found that the risk of mortality from CVD, cancer and respiratory disease decreases up to a threshold of about five portions of fruit and vegetables per d^(6)^. Fruit and vegetable intake has also been linked to mental health benefits. A systematic review of fruit and vegetable intake and mental health found an association between fruit and vegetable intake and better mental health in children aged 4–13 years^(9)^. However, it is unknown whether fruit and vegetable consumption on its own has physical and mental health benefits or whether these individuals have a healthier lifestyle in general. There is also the possibility that poor mental health may impact engagement in healthier dietary intake.

Improving the diet of children is particularly important as poor diet in childhood has been associated with adult health problems such as obesity^(10)^, CVD^(11)^ and diabetes^(12)^. There is evidence that dietary habits acquired in childhood carry on into adulthood^(13)^ and that adults prefer to eat foods that they ate as children^(14)^. Therefore, early intervention in children’s diet will result in health benefits not only in childhood but also when they reach adulthood.

Previous research has shown that parents’ dietary habits, availability, accessibility and preferences are correlated with fruit and vegetable intake in children^(15,16)^. However, Bere and Klepp (2005) also found that past intake was the strongest predictor of future fruit and vegetable intake which supports the importance of targeting healthy eating habits early^(15)^. Nepper and Chai (2016) who carried out interviews with parents of 6–12-year-olds identified the following themes as barriers to their child’s fruit and vegetable consumption: parents’ time, cost of fruit and vegetables, children asking for junk food, children being picky eaters and their child’s early exposure to unhealthy eating^(17)^.

Underpinning interventions with psychological theory have been effective in bringing about behaviour change^(18)^. The Behaviour Change Wheel (BCW) framework which incorporates the Capability, Opportunity, Motivation and Behaviour (COM-B) model (Fig. 1)^(19)^ ascertains that for behaviour to occur there must be physical and psychological Capability, social and physical Opportunity, and reflective (thought processes) and automatic Motivation (intrinsic responses such as desires and impulses). To change a behaviour, one or more of the components need to be changed. The BCW framework consists of eight steps for behaviour change; however, this study will focus on the first five steps to identify the behaviours that are associated with increasing children’s fruit and vegetable consumption and will suggest possible intervention functions to target this behaviour. The COM-B model will be mapped out onto the Theoretical Domains Framework (TDF) (Fig. 1)^(20)^. The TDF consists of fourteen domains across numerous theoretical constructs. These domains are ‘Knowledge’, ‘Skills’, ‘Social/Professional Role and Identity’, ‘Beliefs about Capabilities’, ‘Optimism’, ‘Beliefs about Consequences’, ‘Reinforcement’, ‘Intentions’, ‘Goals’, ‘Memory, Attention and Decision Processes’, ‘Environmental Context and Resources’, ‘Social Influences’, ‘Emotion’ and ‘Behavioural Regulation’. The TDF can be used to provide a framework of questions that will give an understanding of increasing children’s fruit and vegetable consumption. The components of the TDF that are identified as being pertinent to the behaviour of interest can then be mapped onto intervention functions and corresponding evidence-based behaviour change techniques. Most research on the application of the COM-B and the TDF to understand dietary behaviour has focused on adult populations^(21)^ or adults with pre-existing health conditions^(22)^. There is limited research on applying the COM-B and TDF to understand children’s dietary behaviour. Porter et al.’s. (2023) application of the COM-B and TDF focused on the influences on families feeding practices in using effective strategies to increase their child eating vegetables^(23)^. Using semi-structured interviews with parents of 2–4 years of age found that five of the six components of the COM-B were identified as components for change, supporting previous research that child food preferences, cost and food waste are barriers. In addition to previous findings, they identified that some feeding practices did not align with parental perceptions, and that knowledge regarding their child’s eating behaviour enabled them to persevere with feeding techniques. Specifically, they assessed that education, persuasion, training, enablement, modelling and environmental restructuring as suitable intervention functions. This study aims to build on this research in two ways: it will measure and address fruit and vegetables as both fruit and vegetables intake have been associated with health benefits^(5,6,9)^, and it will also focus on increasing fruit and vegetable consumption in children of primary school age.

**Figure 1.**
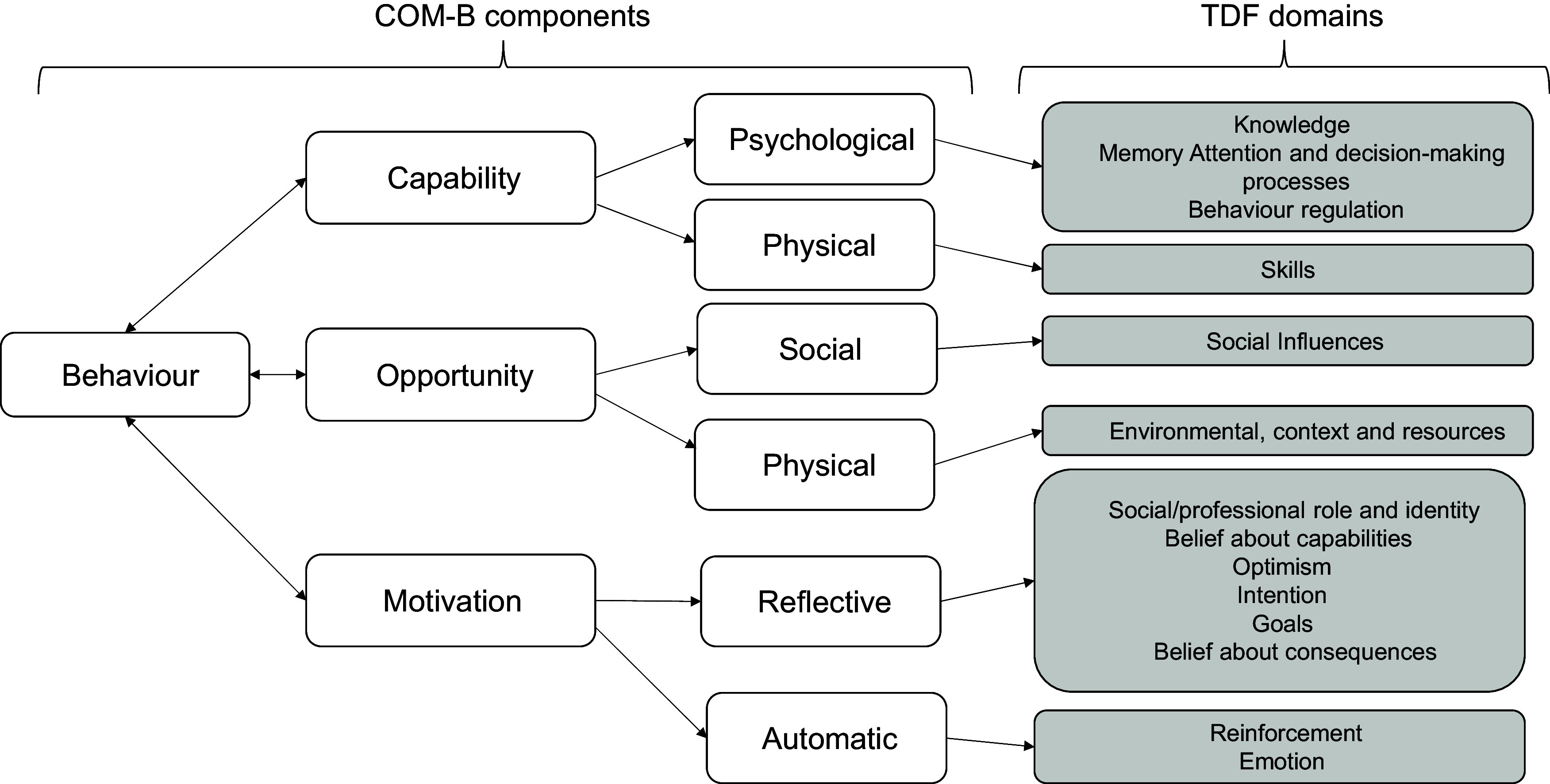
COM-B model components mapped onto the corresponding TDF domains. COM-B, Capability, Opportunity, Motivation and Behaviour; TDF, Theoretical Domains Framework.

## Rationale

This study utilised the BCW and TDF to explore behaviours related to increasing primary school-aged children’s fruit and vegetable consumption from a parent’s perspective. The BCW and TDF are effective models that have been applied to explore diet-related behaviour^(21,23)^. However, as far as the authors are aware, they have not yet been applied to increasing fruit and vegetable consumption in this age group.

Latest figures in England indicate that 9·2 % of reception aged children (4–5 years) are living with obesity. This increases to 22·7 % of children by the time they leave primary school (aged 10–11 years)^(24)^. As obesity correlates with low intake of fruit and vegetables^(7)^, targeting dietary interventions at primary school-aged children is critical. It is important to increase children’s fruit and vegetable consumption due to the impact that it has on their current and future physical and mental health. This study will specifically target children who are not eating the recommended amount of fruit and vegetables to help aid intervention development for this at-risk group. As primary school-aged children do not control the purchasing and availability of fruit and vegetables, this study is aimed at parental behaviours. Parents have a strong influence in shaping their child’s eating habits^(25)^, and research highlights the importance of targeting parental feeding behaviours^(26)^.

This study used the COM-B model to identify and explore parental factors that are associated with their child’s fruit and vegetable consumption, with findings being used to identify intervention functions that are likely to lead to the behaviour change of increased fruit and vegetable consumption in children. Specifically, the objectives were to explore parental perceived capability, opportunity and motivation to increase their child’s fruit and vegetable consumption and determine parental barriers and facilitators using the BCW and TDF to increase their child’s fruit and vegetable consumption and identify intervention functions that are likely to increase fruit and vegetable consumption in children.

## Methods

### Design

A qualitative design was used to elicit an understanding of behaviours surrounding COM-B on increasing fruit and vegetable consumption in primary school-aged children. Capability, Opportunity and Motivation were further divided using the TDF into the fourteen domains to provide a more detailed understanding of the behaviour. An online open-ended survey hosted on Gorilla was administered to capture in-depth information about parents’ thoughts and behaviours around their child’s fruit and vegetable consumption.

### Participants

The ideal suggested sample size for participant-generated text is 10–50 participants^(27)^, and Francis et al., (2004) advise that for an elicitation study using open-ended questionnaires a sample size of 25 is ideal^(28)^. Therefore, to extract enough data from the online surveys, a total of twenty-eight parents, both female (*n* 26) and male (*n* 2) aged between 29 and 51 years, participated in this study. Other qualitative design studies using the COM-B and TDF to understand health behaviours have used a similar number of participants^(21–23)^. Participants consisted of parents of primary school-aged children (children aged between 4 and 11 years). Participants were recruited via email advert through their child’s primary school, via online social media platforms, and to enhance recruitment, via Ulster University’s internal email system. Interested participants were able to access the survey by a link in the advert. Participants were able to complete the survey at any time, and there was no time limit imposed to complete the survey. The online survey included the consent form, the participant information sheet and debrief information. The inclusion criteria for the study were parents over the age of 18 years with a primary school-aged child who were not eating five portions of fruit and vegetables daily and live in the UK. The exclusion criteria were parents of children with food allergies or intolerances and parents of children with a learning disability or a health condition.

### Procedure and materials

Participants were recruited via an advert that contained the link to the online survey. At the start of the online survey, participants were able to read the participant information form and provide consent if they wished to participate. Participants were informed that the study was voluntary and that they were free to withdraw at any time up until submission of their completed survey, that the survey was anonymous, that any personal data provided would be confidential and no identifiable information would be used in the study. Once consent was completed, participants were able to answer the survey questions. The survey consisted of demographic questions to explore any trends in responses, behavioural questions concerning their child’s current fruit and vegetable consumption, and twenty open-ended theoretical questions relating to their child’s fruit and vegetable consumption. The open-ended survey questions were developed with and informed by the COM-B model^(19)^ and then mapped onto each of the domains of the TDF^(29)^ (Table 1). Examples of the questions included ‘To what extent is increasing your child’s fruit and vegetable intake something you usually do?’, ‘Do you know how to increase your child’s fruit and vegetable intake?’ and ‘To what extent would increasing your child’s fruit and vegetable intake be accepted by your friends and family?’. At the end of the survey questions, participants were debriefed and signposted to resources if they had concerns about their child’s fruit and vegetable consumption.

**Table 1.** Open-ended survey questions devised from the COM-B model and Theoretical Domains Framework (TDF)

COM-B	TDF	Question
Psychological capability	Knowledge	Do you know the recommended fruit and vegetable intake for children?
		Why do you think it is recommended that children eat at least a set number of fruit and vegetable portions each day?
Psychological capability	Memory, attention and decision processes	To what extent is increasing your child’s fruit and vegetable intake something you usually do?
		Are you aware of how many fruits and vegetables your child eats per day?
Psychological capability	Behaviour regulation	To what extent do you monitor whether your child is eating enough fruit and vegetables each day?
Physical capability	Skills	To what extent are you confident to prepare fruit and vegetables and to make or cook meals that contain fruit and vegetables?
		Do you know how to increase your child’s fruit and vegetable intake?
Social opportunity	Social influences	Do you yourself eat at least 5 portions of fruit and vegetables each day?
		To what extent do/would your family or friends help or hinder increasing your child’s fruit and vegetable intake?
		To what extent does your child influence whether you provide fruit and vegetables each day?
Physical opportunity	Environmental context and resources	Discuss anything in your work or/and home environment that might help or hinder increasing your child’s fruit and vegetable? e.g. budget and time
Reflective motivation	Social/professional role and identity	To what extent would increasing your child’s fruit and vegetable intake be accepted by your friends and family?
Reflective motivation	Beliefs about capabilities	How difficult/easy would it be for you to increase your child’s fruit and vegetable intake?
		What are the barriers do you think that would hinder increasing your child’s fruit and vegetable intake?
		What do you think would help to increase your child’s fruit and vegetable intake?
Reflective motivation	Optimism	To what extent are you confident that any barriers to increasing your child’s fruit and vegetables can be solved?
Reflective motivation	Intention	To what extent do you intend to increase your child’s fruit and vegetable intake?
Reflective motivation	Goals	To what extent would you like to increase your child’s fruit and vegetable intake?
Reflective motivation	Beliefs about consequences	What do you think the benefits are if your child eats more fruit and vegetables?
		What do you think the consequences are if your child is not eating enough fruit and vegetables each day?
Automatic motivation	Reinforcement	Are there incentives for you to increase your child’s fruit and vegetable intake?
Automatic motivation	Emotion	Discuss how you think increasing your child’s fruit and vegetable intake will make you feel?
Is there anything else that we have not discussed that affects your child eating fruit and vegetables?		

COM-B, Capability, Opportunity, Motivation and Behaviour.

### Data analysis

Qualitative data from the online survey were analysed and coded using thematic analysis^(30)^. The researcher (LG) conducted a thorough familiarisation with the dataset and kept a reflective diary throughout the process to ensure a clear overview of the material. Inductive coding was utilised on survey responses with each code identified as either a facilitator or barrier to increasing fruit and vegetable consumption. From the data coded, 10 % was cross-checked (with JD). There was a strong inter-rater agreement (83 %)^(31)^ on coding achieved with differences discussed and resolved. Once the codes had been agreed upon, summative content analysis^(32)^ was applied, where the researcher searched the text for occurrences of codes and frequency counts for each identified code. The TDF domains were judged based on the frequency count of coding for each TDF domain. TDF domains were then ranked-ordered as either a facilitator or as a barrier (see Table 2). From the COM-B and TDF analysis, a ‘behavioural diagnosis’ was formed (see Table 3). This was then mapped onto the nine intervention functions from the BCW^(19)^ namely, ‘Education’, ‘Persuasion’, ‘Incentivisation’, ‘Coercion’, ‘Training’, ‘Restriction’, ‘Environmental restructuring’, ‘Modelling’ and ‘Enablement’.

**Table 2.** Barriers and facilitators in rank order of mentions in relation to parental perspectives of their child’s fruit and vegetable consumption

COM-B	TDF	Rank order	No. of questions	Frequency of mentions related to codes	% of mentions
Facilitators					
Physical capability	Skills	1	2	68	16
Psychological capability	Memory attention and decision processes	2	2	56	13
Psychological capability	Knowledge	3	2	52	12
Reflective motivation	Beliefs about consequences	4	2	52	12
Social opportunity	Social influences	5	3	41	9
Reflective motivation	Goals	6	1	30	7
Reflective motivation	Beliefs about capabilities	7	3	26	6
Automatic motivation	Emotion	8	1	24	6
Reflective motivation	Social/professional role and identity	9	1	23	5
Psychological capability	Behavioural regulation	10	1	19	4
Automatic motivation	Reinforcement	11	1	15	3
Reflective motivation	Optimism	12	1	13	3
Physical opportunity	Environmental context and resources	13	1	10	2
Reflective motivation	Intentions	14	1	9	2
	Total			438	100

COM-B	TDF	Rank order	No. of questions	Frequency of mentions related to codes	% of mentions
Barriers					
Physical opportunity	Environmental context and resources	1	1	79	33
Reflective motivation	Beliefs about capabilities	2	3	52	22
Social opportunity	Social influences	3	3	46	19
Psychological capability	Knowledge	4	2	14	6
Automatic motivation	Reinforcement	5	1	10	4
Psychological capability	Memory attention and decision processes	6	2	10	4
Reflective motivation	Intentions	7	1	7	3
Psychological capability	Behavioural regulation	8	1	6	3
Reflective motivation	Optimism	9	1	6	3
Physical capability	Skills	10	2	6	3
Automatic motivation	Emotion	11	1	2	1
Reflective motivation	Beliefs about consequences	12	2	2	1
Reflective motivation	Social/professional role and identity	13	1	1	0
Reflective motivation	Goals	14	1	0	0
	Total			240	100

COM-B, Capability (C), Opportunity (O), Motivation (M), Behaviour (B); TDF, Theoretical Domains Framework.

Information above the thick line represents the top-mentioned TDF domains. 80 % of mentions fell under these TDF domains.

**Table 3.** Behavioural diagnosis of increasing child’s fruit and vegetable intake using the BCW and COM-B

COM-B	TDF	F/B	Relevance of domain	Intervention function
Physical capability	Skills	F	Most parents reported that they knew how to increase their child’s fruit and vegetable consumption and they were confident with preparing FV. Therefore not relevant.	n/a
Psychological capability	Knowledge	F/B	Most parents knew the fruit and vegetable recommendations; however, they did not know childhood diet impacts adult diet. Therefore relevant.	Education
Psychological capability	Memory attention and decision processes	F	Most parents were aware of their child’s fruit and vegetable intake and made attempts to increase it. Therefore not relevant.	n/a
Social opportunity	Social influences	F/B	Close family give unhealthy foods to child which hinders attempts to increase fruit and vegetables. Negative role modelling of unhealthy food by close family. Therefore relevant.	Restriction, Environmental restructuring, Modelling, Enablement
Physical opportunity	Environmental context and resources	B	Parents mentioned often that that time and cost were a barrier to increasing their child’s fruit and vegetable consumption. Therefore relevant.	Training, Restriction, Environmental restructuring, Enablement
Reflective motivation	Beliefs about consequences	F	Parents knew about some of the benefits of their child in 5 portions of fruit and vegetables, but only a few mentioned mental health and future health. Therefore relevant.	Education, Persuasion, Modelling
Reflective motivation	Goals	F	Most parents reported that they wanted to increase their child’s fruit and vegetable intake. Therefore not relevant.	n/a
Reflective motivation	Beliefs about capabilities	F/B	Most parents mentioned that their child’s food preferences/refusal affected their ability to increase their fruit and vegetable consumption. Alternative fruit and vegetable presentation was felt would increase fruit and vegetable consumption. Therefore relevant.	Education, Persuasion, Modelling, Enablement
Automatic motivation	Emotion	F	Most parents felt that increasing their child’s fruit and vegetable consumption would increase positive feelings for them. Therefore not relevant.	n/a

BCW, Behaviour Change Wheel; COM-B, Capability (C), Opportunity (O), Motivation (M), Behaviour (B); TDF, Theoretical Domains Framework; F, identified as a facilitator; B, identified as a barrier.

## Results

In total, twenty-eight participants participated in the study. Of the twenty-eight responses that completed the survey, one response contained missing data to ten out of the thirty-five questions (28·57 % missing). However, all data have been included in the analysis. Participants were both male (*n* 2; 7 %) and female (*n* 26; 93 %) with an age range of 29–51 years and an average age of 40·14 years (sd = 4·98). The children were both male (*n* 17; 60 %) and female (*n* 11; 40 %) with an average age of 8·12 (sd = 2·06) years. Of the participants’ children, 11 % were in receipt of free school meal entitlement. All participants reported that they purchased fresh fruit and vegetables with 71 % of participants purchasing frozen and 21 % of participants purchasing processed fruit and vegetables. Parents reported that their children were eating on average 3·07 portions of fruit and vegetables per d with slightly more fruit portions being eaten than vegetables (see Table 4 for summary of participant characteristics).

**Table 4.** Summary of participant characteristics (*n* 28)

Characteristic	*n*	%
Gender of parent		
Male	2	7
Female	26	93
Age of parent (years)		
29–39	12	43
40–51	16	57
Education of parent*		
No formal qualifications	0	0
GCSE, GCE O level or equivalent	2	7
GCE A level, Advanced Senior Certificate	1	4
Further education	3	11
Higher education	1	4
Degree level or higher	21	75
Uptake of free school meals		
Yes	3	11
No	25	89
Gender of child		
Male	17	61
Female	11	39
Age of child (years)		
4–7	8	29
8–11	20	71
Number of children in household		
1–2	17	61
3–4	11	39
Purchasing behaviour		
Fresh	28	100
Frozen	20	70
Processed	6	20
Portions of fruit and vegetables eaten	Average	
Portions of fruit	1·71	
Portions of vegetables	1·36	
Portions of fruit and vegetables	3·07	

* Higher education, BTEC (Higher), BEC, TEC, HNC, HND; further education, BTEC (Further/National), ONC, OND.

Table 2 represents the rank order of the barriers and facilitators of the COM-B and TDF domains relating to parental perspectives on their children’s fruit and vegetable consumption. All reports of barriers and facilitators to their child’s fruit and vegetable consumption were able to be mapped onto the TDF and COM-B. Of these, 65 % of mentions were identified to be a facilitator and 35 % were identified as a barrier. The TDF domains above the thick black line represent the key reported TDF domains and corresponding COM-B components which account for over 80 % of data. These cut of figures have been used in previous studies that have implemented the TDF with health-related behaviours^(21,33)^. Of the facilitators, 80 % were captured by eight of the TDF domains: ‘skills’, ‘memory, attention and decision processes’, ‘knowledge’, ‘beliefs about consequences’, ‘social influences’, ‘goals’, ‘belief about capabilities’ and ‘emotion’. Likewise, 80 % of the barriers were captured by four of the TDF domains: ‘environmental, context and resources’, ‘beliefs about capabilities’, ‘social influences’ and ‘knowledge’. See Tables 5 and 6 for key COM-B facilitators and barriers and the sub-themes and quotes relating to these domains. The findings of these key domains and the corresponding COM-B components will be reported in more detail.

**Table 5.** Key COM-B facilitators, sub-themes and quotes from participants regarding increasing their child’s fruit and vegetable consumption

COM-B	TDF	Sub-theme	Quotes
Physical capability	Skills	1. Confidence in cooking /preparation	‘I am very confident in cooking and preparing fruit and vegetables, and confident to serve them in an appetising manner’ (P27).
		2. Offering and hiding fruit and vegetables	‘keeping trying foods even if he indicated that he didnt like the last time he tried them by saying that tastes change. Disguising vegs by chopping finely in foods. Reward charts’ (P2).
Psychological capability	Memory attention and decision processes	1. Awareness	‘at least two pieces of fruit, sometimes 3… but vegetables sometimes none’ (P13)
		2. Attempts to increase	‘I try my best to offer multiple fruits and veg each day, however, some days this will not happen due to busy lifestyles, choosing convenience over quality when busy, etc’ (P18).
Psychological capability	Knowledge	1. Knowledge of ‘5 a day’. 2 Knowledge of why ‘5 a day’	‘the recommended fruit and vegetable intake for children is 5 portions in total per day’ (P1). ‘5 portions per day minimum, varied and dependent on what types of fruit as some are high in sugar e.g. strawberries’ (P14). ‘To ensure they are taking in a minimum amount of essential nutrients, vitamins and minerals on a daily basis’ (P27)
Reflective motivation	Beliefs about consequences	1. Better health	‘Better overall health which affects their immune system, their oral health (teeth) and also their wellbeing. I think when a child feels good, they will sleep well, perform well at school, be in a good mood etc’ (P1). ‘Better vitamins absorbed, a healthier body as they grow and for their body in the future, less chance of disease i.e. diabetes, cancer, creating healthy eating habits when young will help create healthy eating habits when he is older’ (P14)
		2. Improve concentration	‘My child would have more energy and might be able to concentrate better’ (P5).
Social opportunity	Social influences	1. Family support	‘my mother is very good at getting fruit and veg into my children’ (P25). ‘Some friends bring fruit eg punnet of grapes/ strawberries, when visiting’ (P28)
Reflective motivation	Goals	1. Wants/desire to increase	‘I’d like to increase intake by one portion per day’ (P10)
Reflective motivation	Beliefs about capabilities	1. Alternative presentation of fruit and vegetables	‘Being more creative and making home made food look more exciting’ (P5). ‘I know some people hide vegetables in their meals too. This is something that I could try more’ (5).
Automatic motivation	Emotion	1. Positive	‘I think it would make me feel good, happy and satisfied’ (P1). ‘I’d feel happy with my child consuming natural nutrients’ (P10). ‘happier and more at ease knowing she is getting a better diet’ (P13).

COM-B, Capability (C), Opportunity (O), Motivation (M), Behaviour (B); TDF, Theoretical Domains Framework.

**Table 6.** Key COM-B barriers, sub-themes and quotes from participants regarding increasing their child’s fruit and vegetable consumption

COM-B	TDF	Sub-theme	Themes
Physical opportunity	Environmental context and resources	1. Time	‘School and having a busy life: sometimes in the evening due to hobbies we have to eat on the go, or have a quick dinner - usually those times the vegetables offered are reduced’ (P1).
		2. Cost	‘Fruit will run out quickly sometimes so maybe we wont have time to go down to the shop to replenish, or it will go off quickly. Fruit and vegetables can be more expensive than sugary snacks where there are likely to be better deals’ (P14). ‘with the expense of fresh foods, if fruit/veg sits in the fridge and goes off, I sometimes feel more reluctant to buy it on a subsequent food shop’ (P18).
Reflective motivation	Beliefs about capabilities	1. Food preferences/refusal	‘they just like what they like and have always been reluctant to try new foods’ (8). ‘his strong refusal - gag reflex on trying new foods that he doesnt want to try’ (P11). ‘His aversion to trying new foods, he gets scared to try anything that looks green or he hasn’t tried before’ (P14). ‘my child refuses to eat vegetables. We try bit but he just refuses to eat them’ (P13).
Social opportunity	Social influences	1. Negative role modelling	‘we dont eat a healthy balanced diet and this reflects on my sons choices’ (P16).
		2. Family providing unhealthy food	‘The only person who could hinder is their Grandad but he doesn’t live within our household. He lives next door - and often offers chocolate/sweets to the children as snacks’ (P1). ‘Grandparents tend to have alot of sugary snacks in their houses like biscuits, cakes, ice cream. His dad will also tend to buy sweets and bring into the house for himself which our son will ask for so that doesn’t help as he would sometimes rather eat these then fruit’ (P14).
Psychological capability	Knowledge	1. Lack of knowledge of childhood eating habits and future eating habits.	‘I think time will be the only solution - as he matures he will gain ability to try new foods’ (P11). ‘Family very much hindered progress with first child. When left there would be given sweets all day’ (P12).

COM-B, Capability (C), Opportunity (O), Motivation (M), Behaviour (B); TDF, Theoretical Domains Framework.

### Capability

#### Psychological capability

Psychological capability was identified as a facilitator and as a barrier to increasing their child’s fruit and vegetable consumption. These facilitators accounted for 25 % of mentions and fell into two of the fourteen TDF domains: ‘knowledge’ and ‘memory, attention and decision processes’. The barriers accounted for 6 % of mentions and were captured under the TDF domain: ‘knowledge’.

##### Knowledge

All but one participant (*n* 27; 96 %) were aware of how many portions of fruit and vegetables were recommended for children, and twenty-six (93 %) participants knew that the recommendations were for health benefits to their child: ‘*Better overall health which affects their immune system, their oral health (teeth) and also their wellbeing*’ (P1). One-quarter of parents (25 %) believed that fruit and vegetable intake would increase with age, for example, one parent reported that ‘*I think time will be the only solution, as he matures, he will gain ability to try new foods*’ (P11).

##### Memory, attention and decision processes

Memory, attention and decision processes were recorded when parents mentioned that they were aware of their child’s fruit and vegetable intake, made attempts to increase it and reported the mental effort of increasing fruit and vegetables. All parents were aware of their child’s fruit and vegetable consumption when they were at home, and 11 % of parents reported that they were unaware of fruit and vegetable consumption when their child was at school or with a caregiver such as a childminder or grandparent. Most participants attempted to try and increase their child’s fruit and vegetable consumption. One parent responded: ‘*I try my best to offer multiple fruits and vegetables each day, however, some days this will not happen due to busy lifestyles, choosing convenience over quality when busy, etc*’ (P18).

#### Physical capability

Physical capability was a key COM-B facilitator in this study for increasing their child’s fruit and vegetable consumption. This facilitator accounted for 16 % of mentions and came under the TDF domain: ‘skills’.

##### Skills

Skills were defined as the ability and competence to prepare and cook fruit and vegetables, along with the knowledge and ability to increase fruit and vegetable consumption. Most parents (89 %) felt confident in cooking and preparing fruit and vegetables, and parents reported that they were offering and encouraging their child to eat fruit and vegetables. For example, one parent reported that they ‘*keep trying foods even if he indicated that he didn’t like (them) the last time he tried them*’ and using ‘*reward charts for trying new fruit and vegetables*’ (P2). However, four participants reported that they did not know how to increase their child’s fruit and vegetable consumption.

### Opportunity

#### Social opportunity

Social opportunity was reported as a facilitator (9 % of mentions) and as a barrier (19 % of mentions) to increasing their child’s fruit and vegetable consumption. The TDF domain that social opportunity falls under is ‘social influences’.

##### Social influences

Social influences were measured in terms of the influences that family and friends have on increasing their child’s fruit and vegetable consumption and the modelling of the desired behaviour. The most reported barriers that were recorded under social influences were that close family members, especially grandparents were giving their child unhealthy foods which hindered their ability to increase fruit and vegetables: ‘*family is still counterproductive. one member actively gives child fizzy drinks, and junk food behind our back, to undermine progress*’ (P12). In half of the participants (53 %), there was negative role modelling of unhealthy food eaten by close family members and the parents not eating five portions of fruit and vegetables themselves, such as one parent reported that ‘*we don’t eat a healthy balanced diet, and this reflects on my son’s choices*’ (P16). Only 46 % of parents were eating five portions of fruit and vegetables daily themselves. Some parents reported that family and friends actually helped to increase their child’s fruit and vegetable consumption.

#### Physical opportunity

Physical opportunity was the most frequently reported COM-B component in this study and was identified as a barrier to increasing fruit and vegetables with 33 % of mentions in the survey.

##### Environmental context and resources

Physical opportunity was defined as anything in the parent’s environment or situation that was associated with increasing their child’s fruit and vegetable consumption such as time, cost and availability. Nearly two-thirds of parents (64 %) mentioned time as a barrier to increasing fruit and vegetables. Parents reported that activities after school that their children are participating in and not being at home long enough affected their ability to offer fruit and vegetables. This was captured in one parent’s comment that ‘*sometimes in the evening due to hobbies we have to eat on the go or have a quick dinner, usually those times the vegetables offered are reduced*’ (P1).

Several parents also reported that fresh fruit and vegetables do not last long and therefore need replenishing throughout the week which is difficult due to busy lifestyles. Almost half (46 %) of parents mentioned that the cost of fruit and vegetables was a barrier to increasing their child’s consumption: ‘*budget would be a big factor in increasing fruit and vegetables. I tend to look for multibuy deals for breakfast and lunches to save money*’ (P5). Food waste of uneaten fruit and vegetables was also reported as a barrier.

### Motivation

#### Reflective motivation

Reflective motivation was identified as a facilitator and as a barrier to increasing their child’s fruit and vegetable consumption. The facilitators accounted for 25 % of mentions and out of the six TDF domains that fall under reflective motivation, three of them were in the top 80 % of mentions. These were ‘beliefs about consequences’, ‘goals’ and ‘beliefs about capabilities’. The barriers accounted for 22 % of mentions and fell under the TDF domain: ‘beliefs about capabilities’.

##### Beliefs about consequences

Beliefs about consequences was coded when parents reported the anticipated outcomes of their children eating the recommended amount of fruit and vegetables each day. This TDF domain was a facilitator in increasing fruit and vegetables and accounted for 12 % of utterances. Parents reported that their children’s physical health, diet, sleep and concentration would be better if their children ate the recommended portions of fruit and vegetables. A few parents mentioned an improvement in mental health and only two parents reported the impact of fruit and vegetables on their child’s future health, for example, one parent reported that a ‘*decreased immunity is the only consequence I can detect (in) them, this may also be caused by other factors*’ (P15).

##### Goals

Goals were coded when parents mentioned in the survey that they want to or try to increase their child’s fruit and vegetable consumption such as ‘*I fully intend to increase her fruit and vegetable intake*’ (P5). The majority of parents (*n* 23; 82 %) reported that they would like to increase their child’s fruit and vegetable intake.

##### Beliefs about capabilities

Beliefs about capabilities was defined by how difficult parents felt that increasing their child’s fruit and vegetable consumption would be and their perceived ability to increase fruit and vegetables. Beliefs about capabilities were reported as a facilitator (6 %) and as a barrier (22 %) to increasing fruit and vegetable consumption. Many parents (64 %) felt that their child’s food preferences and food refusal affected their ability to increase their child’s fruit and vegetable intake: ‘*they just like what they like and have always been reluctant to try new foods*’ (P8). Several parents reported that if they provided an alternative presentation, blended fruit and vegetables and hid them in meals, this would increase their child’s intake. Almost a third (36 %) of parents felt that it would be easy to increase their child’s fruit and vegetable intake, especially if they had more time. Half of parents (50 %) mentioned that it would be difficult for them to increase their child’s fruit and vegetable intake, for example, ‘*I would have to have to put quite a bit of effort into increasing my child’s vegetable intake as they claim to not enjoy the taste of many vegetables*’ (P15).

#### Automatic motivation

Automatic motivation was identified as a facilitator to increasing fruit and vegetables. This component accounted for 6 % of mentions as a facilitator. Out of the two TDF domains that fell under automatic motivation. Emotion was identified as a facilitator and reinforcement was not identified as a key facilitator or a barrier to parents increasing their child’s fruit and vegetables consumption.

##### Emotion

Emotion was measured by parents’ reported feelings on how increasing their child’s fruit and vegetables would make them feel, with 82 % of parents mentioning that increasing their child’s fruit and vegetable intake would result in positive feelings for them. Such as, one parent reported that they would feel ‘*happier because I feel I am helping him learn to be healthier and hopefully he will continue this as he gets older*’ (P14) and another parent stated ‘*I would love my child to eat more fruits and vegetables, it would make meal times calmer and more enjoyable*’ (P24).

### Behavioural diagnosis

Using the BCW to identify intervention functions, the key TDF domains and the corresponding COM-B components for facilitators and barriers were assessed as to whether they are relevant for behaviour change (see Table 3). The intervention functions identified as most likely to change parents increasing their child’s fruit and vegetable consumption are ‘Education’, ‘Persuasion’, ‘Training’, ‘Restriction’, ‘Environmental restructuring’, ‘Modelling’ and ‘Enablement’.

## Discussion

This study sought to apply the BCW and TDF to explore parents’ perspectives increasing their child’s fruit and vegetable consumption. Data from parental responses identified that 80 % of the facilitators came under eight TDF domains (skills; memory, attention, and decision processes; knowledge; beliefs about consequences; social influences; goals; beliefs about capabilities; and emotions) and 80 % of barriers fell under only four TDF domains (environmental, context and resources; beliefs about capabilities; social influences; and knowledge). The results confirmed previous findings that parents’ dietary habits and child’s food preferences^(15,16,23)^, budget and time^(17,23)^, were factors that were associated with increasing their children’s fruit and vegetable intake. Novel findings that were a barrier to increasing their children’s fruit and vegetable intake were incorrect parental beliefs and lack of knowledge.

Parents were knowledgeable about the recommended daily amount of fruit and vegetables for their children and the associated health benefits. Parents were also aware of how many fruits and vegetables their children were eating and knew how to prepare and cook fruit and vegetables, consistent with previous research which found that cooking and preparation skills were associated with a greater consumption of fruit and vegetables^(34)^. This highlights capability as a key facilitator in increasing fruit and vegetable consumption. However, a novel finding from this study was that a quarter of parents (25 %) reported/believed that their child’s fruit and vegetable intake will increase with age regardless. This may explain why only a small number of parents (32 %) intended to increase consumption. Given that considerable research shows that repeated exposure over time can increase fruit and vegetable consumption^(35,36)^, it is possible that parents are confused as to the mechanisms/techniques that increase fruit and vegetable consumption with age. Therefore, it is suggested that future interventions target parental and skills around how to increase fruit and vegetables at home as their child ages. This highlights and supports the importance of targeting fruit and vegetable intake in children whilst they are young, especially considering that dietary behaviours in childhood are more likely to continue into adulthood^(13)^.

Interestingly, it was not only the parents of children entitled to free school meals who reported that cost was a barrier. Almost half of parents surveyed felt that the cost and food waste from uneaten fruit and vegetables hindered their ability to increase their child’s consumption. This finding is supported by the research literature^(17)^. However, recent research has found that if households prioritise healthy eating, such as eating more fruit and vegetables, then they are better able to afford and consume more fruit and vegetables^(37)^. In contrast, a UK study by Dogbe and Revoredo-Giha (2021) suggested that to increase fruit and vegetable consumption by 10 %, the price of fruit needs to decrease by 13 % and vegetables need to decrease by 21 %^(38)^. Therefore, finding more affordable ways to eat fruit and vegetables is essential. Canned (processed) or frozen fruits and vegetables are lower or comparable to fresh fruit and vegetables and are therefore a cost-effective way to meet recommendations^(39)^. With 70 % of parents in this study reporting that they purchase frozen and only 20 % of parents reporting that they purchased processed fruit and vegetables, this may be a cost-effective alternative to increase their child’s consumption.

Close family members, particularly grandparents, were hindering increasing fruit and vegetable intake by offering unhealthy foods; this finding is consistent with previous research where grandparents have been found to negatively influence the dietary intake of their grandchildren^(40)^. However, other research found evidence that grandparents generally provide healthy foods^(41)^. Given that about 50 % of grandparents are providing some proportion of childcare^(42)^, strategies and interventions need to also be aimed beyond the home environment.

Parents were able to state the benefits of their child eating the recommended amount of fruit and vegetables; however, what this study found was that parents were not aware of the mental health benefits of fruit and vegetable consumption^(9)^ nor the impact that their child’s fruit and vegetable consumption could have on their future health^(10)^. Research indicates that one in five children have a probable mental health disorder^(43)^ and that incidences of type 2 diabetes is increasing in children^(44)^. Therefore, it is crucial that early parental dietary interventions are prioritised. It is recommended that there is a focus on expanding parental knowledge to lead to a greater understanding of the many benefits of their children eating five portions of fruit and vegetables daily which may influence their intentions and attempts to increase intake.

The ‘behavioural diagnosis’ produced in this study indicates that five out of the six COM-B components are potential areas for change. The TDF domains that were deemed relevant are knowledge, social influences, environmental context and resources, beliefs about consequences, and beliefs about capabilities. The intervention functions relating to these domains are Education, Persuasion, Training, Restriction, Environmental restructuring, Modelling and Enablement. The BCW provides a systematic approach to behaviour change, and further research is recommended to identify appropriate behaviour change techniques, modes of delivery and policy categories that best target the intervention areas identified in this study^(19)^.

### Limitations

The study was undertaken with a small sample size of parents (*n* 28); however, this is in line with previous dietary studies that incorporated the COM-B model and TDF^(21–23)^. Most parents in the sample were educated to at least a degree level (75 %), and only 11 % were entitled to free school meals. In 2023, 23·8 % school-aged children in the UK were eligible for free school meals^(45)^. This would suggest that the participants in this study were of higher socio-economic status than the population as a whole. As the ethnicity of parents was not gathered, it is unknown whether the sample included parents from a range of cultural backgrounds. Therefore, the findings in this study may not be generalised to the general population. However, the aim of the study was to gain a greater depth of understanding of parental perspectives.

As the majority of participants were mothers, the results may not be representative of fathers’ perspectives. However, it is likely that mothers will engage most on this issue as they are generally the primary caregivers. This has been found in previous research that aimed to understand children’s dietary behaviour^(46)^. It is therefore suggested that future research aims to gather data from a greater diverse population representative of socio-economic status, level of education, ethnicity and gender. Researcher subjectivity may have been a limitation in the study; however, the researcher aimed to minimise this by keeping a reflective diary throughout analysis, and codes were cross-checked with a second individual (JD) who is experienced in qualitative research.

Lastly, the data collection method used in the study may have limitations such as the use of self-report and using an open-ended survey as opposed to focus groups or interviews. Self-reporting can be affected by recall and memory and influenced by various factors such as time, emotions and motivation^(47)^. These factors could have affected reliability of parental responses. While focus groups and interviews can increase the desire for social acceptability^(48)^, this study was anonymous and online; therefore, participants would have been less likely to respond to social pressures.

### Strengths

The COM-B is a successful theory-based model for understanding behaviour and has been utilised extensively in dietary change behaviour^(21,23)^. Using the BCW allowed a systematic approach to identifying the barriers and facilitators to enable a ‘behavioural diagnosis’. To the researcher’s knowledge, this study was the first to develop a ‘behavioural diagnosis’ using the BCW and TDF to identify what needs to change to increase children’s fruit and vegetable consumption in primary school-aged children and link this to intervention functions to inform future intervention development.

### Conclusion

The findings from this study using the BCW and TDF identified that for fruit and vegetable intake behaviour change to occur that ‘knowledge’, ‘social influences’, ‘environmental context and resources’, ‘beliefs about consequences’ and ‘beliefs about capabilities’ need to change. From the results obtained, five out of the six components of the COM-B model are required to increase fruit and vegetable consumption. The findings from this study recommend using the intervention functions identified to inform behaviour change techniques, modes of delivery and policy categories. As parents have a pivotal role in their child’s dietary intake, it is suggested that behaviour change techniques address the home environment as well as the wider home environment for successful behaviour change to increase children’s fruit and vegetable consumption.

## References

[1] NHS (2022) Why 5 a Day? https://www.nhs.uk/live-well/eat-well/5-a-day/why-5-a-day/#:∼:text=Fruit%20and%20vegetables%20are%20a,your%20risk%20of%20bowel%20cancer (accessed May 2023).

[2] World Health Organisation (2020) Healthy Diet. https://www.who.int/news-room/fact-sheets/detail/healthy-diet (accessed May 2023).

[3] NHS Digital (2019) Fruit and Vegetables. http://healthsurvey.hscic.gov.uk/data-visualisation/data-visualisation/explore-the-trends/fruit-vegetables.aspx (accessed April 2023).

[4] Foods Standard Agency (2019) National Diet and Nutrition Survey (NDNS RP): Results for Years 5 to 9 (combined) of the Rolling Programme for Northern Ireland (2012/13–2016/17) and time trend and income analysis (Years 1 to 9; 2008/09–2016/17). https://www.food.gov.uk/sites/default/files/media/document/national-diet-and-nutrition-survey-northern-ireland-y5-9-full-report_2.pdf (accessed June 2023).

[5] Aune D , Giovannucci E , Boffetta P et al. (2017) Fruit and vegetable intake and the risk of cardiovascular disease, total cancer and all-cause mortality—a systematic review and dose-response meta-analysis of prospective studies. Int J Epidemiol 46, 1029–1056. 28338764 10.1093/ije/dyw319 PMC5837313

[6] Wang DD , Li Y , Bhupathiraju SN et al. (2021) Fruit and vegetable intake and mortality: results from 2 prospective cohort studies of US men and women and a meta-analysis of 26 cohort studies. Circulation 143, 1642–1654. 33641343 10.1161/CIRCULATIONAHA.120.048996 PMC8084888

[7] Mekonnen T , Tariku A & Abebe SM (2018) Overweight/obesity among school aged children in Bahir Dar City: cross sectional study. Ital J Pediatr 44, 17. 29361952 10.1186/s13052-018-0452-6 PMC5781282

[8] Sdona E , Ekström S , Andersson N et al. (2022) Fruit, vegetable and dietary antioxidant intake in school age, respiratory health up to young adulthood. Clin Exp Allergy 52, 104–114. 34549838 10.1111/cea.14020

[9] Guzek D , Głąbska D , Groele B et al. (2020) Role of fruit and vegetables for the mental health of children: a systematic review. Rocz Panstw Zakl Hig 71, 5–13. 32227779 10.32394/rpzh.2019.0096

[10] Rampelli S , Guenther K , Turroni S et al. (2018) Pre-obese children’s dysbiotic gut microbiome and unhealthy diets may predict the development of obesity. Commun Biol 1, 222. 30534614 10.1038/s42003-018-0221-5 PMC6286349

[11] Kaikkonen JE , Mikkilä V , Magnussen CG et al. (2013) Does childhood nutrition influence adult cardiovascular disease risk? Insights from the Young Finns Study. Ann Med 45, 120–128. 22494087 10.3109/07853890.2012.671537

[12] Jääskeläinen P , Magnussen CG , Pahkala K et al. (2012) Childhood nutrition in predicting metabolic syndrome in adults: the cardiovascular risk in Young Finns Study. Diabetes Care 35, 1937–1943. 22815293 10.2337/dc12-0019 PMC3425009

[13] Movassagh EZ , Baxter-Jones ADG , Kontulainen S et al. (2017) Tracking dietary patterns over 20 years from childhood through adolescence into young adulthood: the Saskatchewan pediatric bone mineral accrual study. Nutrients 9, 990. 28885565 10.3390/nu9090990 PMC5622750

[14] Wadhera D , Capaldi Phillips ED , Wilkie LM et al. (2015) Perceived recollection of frequent exposure to foods in childhood is associated with adulthood liking. Appetite 89, 22–32. 25616213 10.1016/j.appet.2015.01.011

[15] Bere E & Klepp KI (2005) Changes in accessibility and preferences predict children’s future fruit and vegetable intake. Int J Behav Nutr Phys Act 2, 15. 16216124 10.1186/1479-5868-2-15 PMC1262749

[16] Wolnicka K , Taraszewska A , Jaczewska-Schuetz J et al. (2015) Factors within the family environment such as parents’ dietary habits and fruit and vegetable availability have the greatest influence on fruit and vegetable consumption by Polish children. Public Health Nutr 18, 2705–2711. 26416288 10.1017/S1368980015000695 PMC10271367

[17] Nepper MJ & Chai W (2016) Parents’ barriers and strategies to promote healthy eating among school-age children. Appetite 103, 157–164. 27090341 10.1016/j.appet.2016.04.012

[18] Painter JE , Borba CP , Hynes M et al. (2008) The use of theory in health behavior research from 2000 to 2005: a systematic review. Ann Behav Med 35, 358–362. 18633685 10.1007/s12160-008-9042-y

[19] Michie S , Atkins L & West R (2015) The Behaviour Change Wheel: A Guide to Designing Interventions. London: Silverback Publishings.

[20] Cane J , O’Connor D & Michie S (2012) Validation of the theoretical domains framework for use in behaviour change and implementation research. Implement Sci 7, 37. 22530986 10.1186/1748-5908-7-37 PMC3483008

[21] Timlin D , McCormack JM & Simpson EE (2021) Using the COM-B model to identify barriers and facilitators towards adoption of a diet associated with cognitive function (MIND diet). Public Health Nutr 24, 1657–1670. 32799963 10.1017/S1368980020001445 PMC8094434

[22] Keaver L , Douglas P & O’Callaghan N (2023) Perceived barriers and facilitators to a healthy diet among cancer survivors: a qualitative exploration using the TDF and COM-B. Diet 2, 123–139.

[23] Porter L , Chater AM , Haycraft E et al. (2023) Role-model, reoffer, reward: a thematic analysis and TDF mapping of influences on families’ use of evidence-based vegetable feeding practices. Appetite 189, 106764. 37442525 10.1016/j.appet.2023.106764

[24] NHS Digital (2023) National Child Measurement Programme, England, 2022/23 School Year. https://digital.nhs.uk/data-and-information/publications/statistical/national-child-measurement-programme/2022-23-school-year (accessed July 2024).

[25] Scaglioni S , Salvioni M & Galimberti C (2008) Influence of parental attitudes in the development of children eating behaviour. Br J Nutr 99, S22–S25. 18257948 10.1017/S0007114508892471

[26] Mahmood L , Flores-Barrantes P , Moreno LA et al. (2021) The influence of parental dietary behaviors and practices on children’s eating habits. Nutrients 13, 1138. 33808337 10.3390/nu13041138 PMC8067332

[27] Braun V & Clarke V (2013) Successful Qualitative Research: A Practical Guide for Beginners. London: Sage.

[28] Francis J , Eccles MP , Johnston M et al. (2004) Constructing Questionnaires Based on the Theory of Planned Behaviour: A Manual for Health Services Researchers. https://openaccess.city.ac.uk/id/eprint/1735/1/TPB%20Manual%20FINAL%20May2004.pdf (accessed June 2023).

[29] Atkins L , Francis J , Islam R et al. (2017) A guide to using the Theoretical Domains Framework of behaviour change to investigate implementation problems. Implement Sci 12, 77. 28637486 10.1186/s13012-017-0605-9 PMC5480145

[30] Braun V & Clarke V (2022) Thematic Analysis: A Practical Guide. London: Sage.

[31] McHugh ML (2012) Interrater reliability: the kappa statistic. Biochem Med 22, 276–282. PMC3900052 23092060

[32] Hsieh HF & Shannon SE (2005) Three approaches to qualitative content analysis. Qual Health Res 15, 1277–1288. 16204405 10.1177/1049732305276687

[33] Lake AJ , Browne JL , Rees G et al. (2017) What factors influence uptake of retinal screening among young adults with type 2 diabetes? A qualitative study informed by the theoretical domains framework. J Diabetes Complications 31, 997–1006. 28363730 10.1016/j.jdiacomp.2017.02.020

[34] Hanson AJ , Kattelmann KK , McCormack LA et al. (2019) Cooking and meal planning as predictors of fruit and vegetable intake and BMI in first-year college students. Int J Environ Res Public Health 16, 2462. 31373293 10.3390/ijerph16142462 PMC6679210

[35] Appleton KM , Hemingway A , Rajska J et al. (2018) Repeated exposure and conditioning strategies for increasing vegetable liking and intake: systematic review and meta-analyses of the published literature. Am J Clin Nutr 108, 842–856. 30321277 10.1093/ajcn/nqy143 PMC6186211

[36] Nekitsing C , Blundell-Birtill P , Cockroft JE et al. (2018) Systematic review and meta-analysis of strategies to increase vegetable consumption in preschool children aged 2–5 years. Appetite 127, 138–154. 29702128 10.1016/j.appet.2018.04.019

[37] Stewart H , Hyman J , Dong D et al. (2021) The more that households prioritise healthy eating, the better they can afford to consume a sufficient quantity and variety of fruits and vegetables. Public Health Nutr 24, 1841–1850. 33317652 10.1017/S1368980020004929 PMC10195601

[38] Dogbe W & Revoredo-Giha C (2021) Nutritional and environmental assessment of increasing the content of fruit and vegetables in the UK diet. Sustainability 13, 1076.

[39] Miller SR & Knudson WA (2014) Nutrition and cost comparisons of select canned, frozen, and fresh fruits and vegetables. Am J Lifestyle Med 8, 430–437.

[40] Young KG , Duncanson K & Burrows T (2018) Influence of grandparents on the dietary intake of their 2–12-year-old grandchildren: a systematic review. Nutr Diet 75, 291–306. 29446218 10.1111/1747-0080.12411

[41] Jongenelis MI , Talati Z , Morley B et al. (2019) The role of grandparents as providers of food to their grandchildren. Appetite 134, 78–85. 30579879 10.1016/j.appet.2018.12.022

[42] Hank K & Buber I (2009) Grandparents caring for their grandchildren: findings from the 2004 survey of health, ageing, and retirement in Europe. J Fam Issues 30, 53–73.

[43] NHS England (2023) News: One in Five Children and Young People had a Probable Mental Disorder in 2023. https://www.england.nhs.uk/2023/11/one-in-five-children-and-young-people-had-a-probable-mental-disorder-in-2023/ (accessed September 2024).

[44] RCPCH (2020) Diabetes. https://stateofchildhealth.rcpch.ac.uk/evidence/long-term-conditions/diabetes/ (accessed September 2024).

[45] GOV UK (2023) Schools, Pupils and their Characteristics. https://explore-education-statistics.service.gov.uk/find-statistics/school-pupils-and-their-characteristics (accessed August 2023).

[46] Kim HS , Park J , Ma Y et al. (2019) What are the barriers at home and school to healthy eating?: overweight/Obese child and parent perspectives. J Nurs Res 27, e48. 30958391 10.1097/jnr.0000000000000321 PMC6752691

[47] Ravelli MN & Schoeller DA (2020) Traditional self-reported dietary instruments are prone to inaccuracies and new approaches are needed. Front Nutr 7, 90. 32719809 10.3389/fnut.2020.00090 PMC7350526

[48] Acocella I (2012) The focus groups in social research: advantages and disadvantages. Qual Quant 46, 1125–1136.

